# Asymmetric paralog evolution between the “cryptic” gene *Bmp16* and its well-studied sister genes *Bmp2* and *Bmp4*

**DOI:** 10.1038/s41598-019-40055-1

**Published:** 2019-02-28

**Authors:** Nathalie Feiner, Fumio Motone, Axel Meyer, Shigehiro Kuraku

**Affiliations:** 1 Department of Biology, University of Konstanz, Konstanz, Germany; 2 Department of Zoology, University of Oxford, Oxford, United Kingdom; 3 Department of Biology, Lund University, Lund, Sweden; 4 Phyloinformatics Unit, RIKEN Center for Life Science Technologies, Kobe, Japan; 5 Graduate School of Science and Technology, Kwansei Gakuin University, Sanda, Japan; 6 Laboratory for Phyloinformatics, RIKEN Center for Biosystems Dynamics Research, Kobe, Japan

## Abstract

The vertebrate gene repertoire is characterized by “cryptic” genes whose identification has been hampered by their absence from the genomes of well-studied species. One example is the *Bmp16* gene, a paralog of the developmental key genes *Bmp2* and -*4*. We focus on the *Bmp2/4/16* group of genes to study the evolutionary dynamics following gen(om)e duplications with special emphasis on the poorly studied *Bmp16* gene. We reveal the presence of *Bmp16* in chondrichthyans in addition to previously reported teleost fishes and reptiles. Using comprehensive, vertebrate-wide gene sampling, our phylogenetic analysis complemented with synteny analyses suggests that *Bmp2*, -*4* and -*16* are remnants of a gene quartet that originated during the two rounds of whole-genome duplication (2R-WGD) early in vertebrate evolution. We confirm that *Bmp16* genes were lost independently in at least three lineages (mammals, archelosaurs and amphibians) and report that they have elevated rates of sequence evolution. This finding agrees with their more “flexible” deployment during development; while *Bmp16* has limited embryonic expression domains in the cloudy catshark, it is broadly expressed in the green anole lizard. Our study illustrates the dynamics of gene family evolution by integrating insights from sequence diversification, gene repertoire changes, and shuffling of expression domains.

## Introduction

*Bmp2/*4 genes have been studied for almost 50 years since the 1970s^1^, but one member of the class, designated as *Bmp16*, was discovered as late as 2009^2^. It was first found in ray-finned fishes (actinopterygians) including zebrafish, and orthologs have since then only been reported in the African coelacanth and the green anole lizard^3^. This ‘patchy’ distribution of *Bmp16* has suggested that its orthologs were lost independently in at least three lineages (amphibians, archosaurs, and mammals)^3^. In contrast, *Bmp2* and -4 genes belong to the core vertebrate gene repertoire and not a single case of gene loss has been documented so far. Phylogenetic investigations suggest that the *Bmp2*, -*4* and -*16* genes originated from an ancestral gene in a whole genome duplication event dating back to an early phase of vertebrate evolution^2,3^.

Only scarce information on the expression profiles of *Bmp16* genes is available, although *Bmp2* and -4 genes have been intensively investigated on diverse levels of interest. *Bmp16* expression patterns have been described in zebrafish^2^, the Coho salmon^4^, the Senegalese sole^5^ and the blunt snout bream^6^. In the developing zebrafish, *bmp16* transcripts are localized in the swim bladder, heart, tail bud, ectoderm of pectoral and median fin folds and gut epithelium. In the Coho salmon, *bmp16* is expressed in ovaries^4^, in the adult Senegalese sole it is expressed in the brain, intestine, heart and branchial arches^5^, and in the blunt snout bream it is expressed in intermuscular bones^6^.

The vertebrate *Bmp2* and -4 genes are amongst the key regulators orchestrating developmental processes, including axis specification^7^. In bony vertebrates (osteichthyans), *Bmp2* and -*4* are involved in various developmental processes, for example the patterning of limb or fin buds, tail bud, heart, sensory placodes (e.g., otic vesicle, retina), gut-associated mesoderm, branchial arches/pouches, and swim bladder or lungs^8–17^. In teleosts, two paralogs, *bmp2a* and *bmp2b*, are known as orthologs of the non-teleost *Bmp2* gene, while only a single ortholog of the *Bmp4* gene has been reported so far. The two *bmp2* duplicates are derived from the teleost-specific genome duplication (TSGD)^18,19^. Cyclostomes are placed in key phylogenetic positions in vertebrate evolution and deserve particular attention in reconstructing the evolution of vertebrate gene families. The hitherto described repertoire of Bmp2/4/16-related genes in cyclostomes consists of three paralogs in the sea lamprey *Petromyzon marinus* designated as *Bmp2/4-A*, *Bmp2/4-B* and *Bmp2/4-C* ^20^ (the nomenclature reflects their identification prior to the discovery of *Bmp*16 genes). Orthologies between these three cyclostome genes and any individual jawed vertebrate (gnathostome) subtype have proven to be difficult to establish^2^, which is common to relationships between cyclostome and gnathostome genes^21^.

The pleiotropic functions of *Bmp2/4/*16 genes necessitate precise regulation and modulation to ensure specificity of the conveyed cellular signal. BMP proteins function as morphogens that are secreted into the extracellular matrix and transmit signals between cells by binding and activating cell surface receptors^22^. One way of signal modulation is sequential activation of BMP2/4 precursor proteins through proteolytical cleavage. Two cleavage sites (S1 and S2) have been described for BMP2/4 proteins, and only proteins cleaved at both sites are fully active and able to convey long-range signals^23^. Another level of regulation in Bmp signalling is receptor binding. After cleaved BMP2/4 proteins are secreted, they bind cell surface receptors as homo- or heterodimers, with heterodimers being more active than homodimers^24^. Dimerization depends on a set of seven cysteine residues at the C-terminus of the mature BMP protein^25,26^. It is currently not fully understood to what extent these structural characteristics described for BMP2/4 proteins also apply to BMP16 proteins, although there is evidence that zebrafish *bmp2a*, -*2b*, -*4* and -16 are all able to activate the BMP-signalling pathway *in vitro*^3^.

In this study, we focused on the *Bmp2/4/16* group of genes as a test case to study recurrent patterns of gene family evolution and specifically to ask how genes can get lost and to reconstruct the fates of paralogues following gen(om)e duplications. Detailed knowledge of *Bmp2* and -*4* genes provides the comparative framework needed to put these insights into the evolution of the *Bmp16* gene gained from this study into context. We provide a detailed scenario for the evolution of *Bmp16* expression profiles and describe patterns of evolutionary rates, secondary gene losses and a characterization of broader genomic environments containing *Bmp2*, -*4* and -*16* genes, contributing to a novel perspective on the dynamics of gene family evolution.

## Results

### Survey of *Bmp2/4/16* homologs across vertebrates

To obtain an inventory of *Bmp2/4/16* homologs present in the genomes of extant vertebrates, we used RT-PCR screening, RNA sequencing (RNA-seq) and exhaustive database searches. We performed tBlastn searches against the ‘wgs’ database containing a recently released genome assembly of the inshore hagfish *Eptatretus burgeri* (NCBI Assembly ID, GCA_900186335.2). This revealed the existence of three previously unidentified *Bmp2/4/16* homologs, designated *Bmp2/4/16-A*, -*B* and -*C* (Supplementary File 1). Degenerate RT-PCR using cDNA derived from brain tissue of the same species identified a sequence that is highly similar (four base pair differences translating to four amino acid changes) to the *Bmp2/4/16-A* sequence identified in the genome assembly of *E*. *burgeri*. Because of the high similarity, the sequence obtained from our cDNA cloning was not included in the downstream analyses, and for *E*. *burgeri*, only the three *Bmp2/4/16* genes identified in the genome sequence were used (see Methods for details).

In cartilaginous fishes (chondrichthyans), database searches identified *Bmp2*, -*4*, and -*16* orthologs with full-length ORFs in the publicly available genome sequences of the whale shark *Rhincodon typus*^27^ (NCBI Assembly ID, GCA_001642345.2), and *Bmp2* and -*4* sequences with full-length ORFs of the elephant fish *Callorhinchus milii*^28^ (NCBI Assembly ID, GCA_000165045.2). Note that the *R*. *typus Bmp16* gene in the NCBI database is labelled as ‘Bmp2-like’ by its systematic annotation pipeline. In the RNA-seq data of cloudy catshark (*Scyliorhinus torazame*) embryos, we identified full-length sequences *of Bmp2*, -*4* and -*16* genes (Supplementary File 1). A fragment of the small-spotted catshark (*Scyliorhinus canicula*) *Bmp16* gene was identified in an expressed sequence tag (EST) archive^29^. By performing 5′-RACE on embryonic *S*. *canicula* cDNA, we obtained a *Bmp16* fragment spanning ~100 amino acids of the 3′-end of the coding sequence. Our search in the additional elasmobranch genome assemblies recently made available^30^ confirmed the retention of *Bmp2*, -*4* and -*16* orthologs as single copies in chondrichthyans.

RACE-based PCRs on cDNA derived from embryonic green anole (*Anolis carolinensis*) produced a *Bmp16* fragment spanning the complete coding sequence (the Ensembl database contains only a truncated sequence, ENSACAG00000004284). Our database mining identified *Bmp16* sequences of the African coelacanth *Latimeria chalumnae*, the spotted gar *Lepisosteus oculatus* and several teleost fishes in the Ensembl database. The NCBI database contained *Bmp16* sequences of the teleost fish species blackspotted livebearer (*Poeciliopsis turneri*), Atlantic salmon (*Salmo salar*) and gilt-head bream (*Sparus aurata*), and of the Burmese python (*Python molurus*), the garter snake (*Thamnophis sirtalis*), brown-spotted pit viper (*Protobothrops mucrosquamatus*), the bearded dragon (*Pogona vitticeps*), and the Japanese gecko (*Gekko japonicus*). In addition, we identified a full-length *Bmp16* transcript of the Madagascar ground gecko (*Paroedura picta*) in the Reptiliomix database^31^, and cloned the cDNA based on this sequence.

In degenerate RT-PCR screens, we obtained Bmp2 cDNA sequences of *Astatotilapia burtoni, Huso dauricus*, a hybrid sturgeon (*H. dauricus* × *Acipenser ruthenus*), *Polypterus senegalus, Raja clavata, S. canicula, S. torazame* and *P. picta, Bmp4* cDNA sequences of *A. burtoni*, a hybrid sturgeon (*H. dauricus* × *A. ruthenus*), *Lepisosteus platyrhincus, Neoceratodus forsteri, P. senegalus, R. clavata, S. canicula, S. torazame* and *P. picta and Bmp16* cDNA of *A. burtoni*. All sequences used in this study are listed in Supplementary Table S1. All newly identified sequences are deposited in EMBL under accession numbers (study accession number, PRJEB25510; accession IDs, LT989953-LT989973).

### Molecular phylogenetic analyses

**Figure 1:**
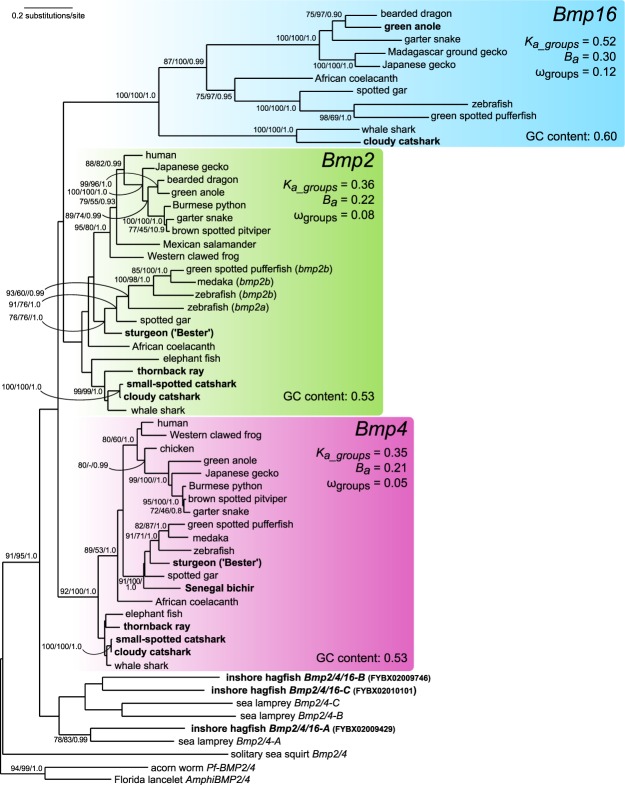
Phylogenetic tree of the *Bmp2/4/16* subgroup. This ML phylogenetic tree shows the relationships within and between the *Bmp2/4/16* orthology groups. Tree inference is based on the JTT + F + I + Γ_4_ model (shape parameter of gamma distribution α = 0.84) and an alignment of 274 amino acids. Support values at nodes are shown in order, bootstrap probabilities in the ML method and in the NJ method, and posterior probabilities in the Bayesian tree inference. Only bootstrap values no less than 70 in the ML analysis are shown. Sequences identified in this study are highlighted in bold. For each group of *Bmp2*, -*4* and -*16* genes, the number of non-synonymous substitutions (*K*_*a_groups*_) and transversions (*B*_*a*_) per non-synonymous site as estimated by RRTree, the non-synonymous/synonymous rate ratio (ω_groups_) as estimated by PAML, and the average GC content calculated by RRTree are given. These were calculated using the full dataset shown in the phylogenetic tree, but with an alignment of codons instead of amino acids (822 nucleotides). See Table S1 for accession IDs of included sequences.

We reconstructed a phylogenetic tree of *Bmp2/4/16* homologs to assess phylogenetic relationships and to infer histories of ancestral gene duplications and losses. Within the jawed vertebrates, the inferred tree exhibited monophyletic grouping of *Bmp4* genes (bootstrap probabilities in maximum-likelihood (ML)/neighbour-joining (NJ)/posterior probability in Bayesian tree inference: 92/100/1.0) and *Bmp16* genes (100/100/1.0), and also *Bmp2* genes were grouped together, although with low support (44/86/-; Fig. 1). In the ML analysis and in the Bayesian tree inference, all jawed vertebrate genes clustered together, while cyclostome *Bmp2/4/16* genes formed a monophyletic sister group. Within cyclostomes, the sea lamprey *Bmp2/4-A* gene clustered with one inshore hagfish gene (78/83/0.99), suggesting a possible orthology between sea lamprey *Bmp2/4-A* and inshore hagfish *Bmp2/4/16-A*. The *Bmp2/4-B and* -*C* genes of the sea lamprey, and the *Bmp2/4/16-B* and -*C* genes of the inshore hagfish formed sister clades which likely indicates that they are species-specific paralogs.

### *Bmp16* evolves twice as fast as Bmp2/4

The phylogenetic tree (Fig. 1) shows that the group of *Bmp16* genes is characterized by longer branches compared to those of *Bmp2* and -*4*. We applied both relative-rate tests (RRTree) and ML-based tests (PAML) to test for evolutionary rate differences between clades and traces of selection acting on protein sequences. Relative-rate tests between groups of genes revealed that the number of non-synonymous substitutions (*K*_*a_groups*_) and transversions (*B*_*a*_) per non-synonymous site (Fig. 1) are significantly different between *Bmp16* and *Bmp2* (*P* value for *K*_*a_groups*_: 1.00 e^−7^; *P* value for *B*_*a*_: 1.00 e^−7^) and between *Bmp16* and *Bmp4* (*P* value for *K*_*a_groups*_: 1.00 e^−7^; *P* value for *B*_*a*_: 1.00 e^−7^), but not between *Bmp2* and *Bmp4* (*P* value for *K*_*a_groups*_: 0.64; *P* value for *B*_*a*_: 0.39). The number of synonymous substitutions (*K*_*s_groups*_) and transitions (*A*_*s*_) per synonymous site could not be estimated, likely due to saturation of synonymous substitutions. A PAML-based estimation of ω values (ratio of nonsynonymous/synonymous substitution rates) reveals that values for the group of *Bmp16* genes (ω_groups_ = 0.12) are approximately twice as large as of the *Bmp2* (ω_groups_ = 0.08) and the *Bmp4* (ω_groups_ = 0.05) group of genes. However, all three values are significantly lower than 1 and are therefore indicative of purifying selection. To obviate the problem of saturated synonymous substitutions, we estimated substitution rates between *Bmp2/4/16* genes in chondrichthyans, squamate and teleost species pairs with relatively recent divergence times (using the program yn00 in PAML). We find that ω_pairwise_ values are elevated 3.5 x in *Bmp16* genes compared to *Bmp2* and -*4* in teleosts and to a lesser extent (1.5X) in squamates, but still in agreement with purifying selection (Table 1). Synonymous substitutions rates do not suggest that background mutation rates between *Bmp2*, -*4* and -*16* genes consistently differ, although teleost *bmp2* genes and chondrichthyan *Bmp16* genes show elevated rates of synonymous substitutions compared to other *Bmp2*, -*4* and -*16* genes analysed (Table 1). A possible explanation for the elevated rates of sequence evolution of *Bmp16* genes could be increased GC-content^32–34^, although we find only limited support for this idea (average GC-content of orthology groups analysed in Fig. 1: *Bmp16*, 0.60, *Bmp2*, 0.53, *Bmp4*, 0.53). Taken together, we find evidence that non-synonymous substitution rates are higher in *Bmp16* genes compared to *Bmp2* and -*4* genes, but no indication of directional selection acting on BMP16 proteins.

**Table 1:** Estimation of differences in evolutionary rates between *Bmp2*, *-4* and -*16* genes.

Species pair	Divergence time	Gene	*K*_*a_pairwise*_^a^	*K*_*s_pairwise*_^b^	ω_pairwise_
*three-spined stickleback : green spotted pufferfish*	110 mya^c^	*bmp2b*	0.035	1.157	0.030
*three-spined stickleback : green spotted pufferfish*	110 mya^c^	*bmp4*	0.029	0.616	0.047
*three-spined stickleback : green spotted pufferfish*	110 mya^c^	*bmp16*	0.104	0.771	0.135
*whale shark : cloudy catshark*	178 mya^d^	*Bmp2*	0.074	0.389	0.190
*whale shark : cloudy catshark*	178 mya^d^	*Bmp4*	0.022	0.600	0.037
*whale shark : cloudy catshark*	178 mya^d^	*Bmp16*	0.157	1.317	0.119
*green anole : bearded dragon*	157 mya^c^	*Bmp2*	0.042	0.589	0.072
*green anole : bearded dragon*	157 mya^c^	*Bmp4*	0.110	0.667	0.165
*green anole : bearded dragon*	157 mya^c^	*Bmp16*	0.146	0.830	0.176

^a^Estimated number of nonsynonymous substitutions per nonsynonymous site.

^b^Estimated number of synonymous substitutions per synonymous site.

^c^Source: http://www.timetree.org/.

^d^Source: Irisarri *et al*.^76^.

### Are *Bmp2*, -*4* and -*16* derived from the 2R-WGD?

**Figure 2:**
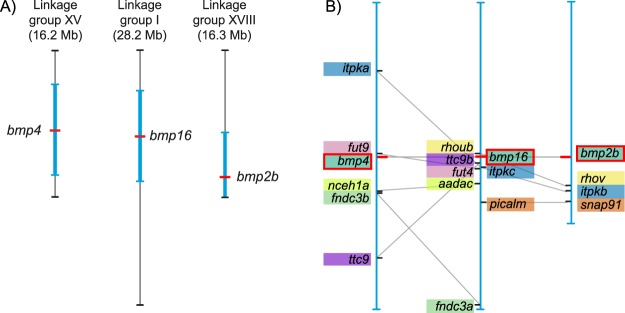
Intra-genomic synteny conservation between regions containing *bmp2b*, -*4* and -*16* genes in the three-spined stickleback genome. (**A**) Overview of three-spined stickleback chromosomes containing *bmp2b*, -*4* and *-16* genes. For each of these three genes, 10 Mb flanking regions (whenever available) are highlighted in blue, and these regions are magnified in B. (**B**) Paralogous relationships of *bmp2b/4/16*-neighbouring genes. Gene pairs and triplets are highlighted with coloured boxes. All genes shown here are derived from duplication events occurring early in vertebrate evolution, i.e. after the split of tunicates, but before jawed vertebrate radiations (see Fig. S1 for gene family trees). Gene-level paralogy between the three chromosomal regions are indicated with grey lines.

The phylogenetic tree (Fig. 1) suggests the origin of jawed vertebrate *Bmp2/4/16* genes in duplications occurring in an early period of vertebrate evolution. However, the exact timing of the duplications remains contested. A reasonable assumption is that the duplications giving rise to *Bmp2/4/16* genes coincided with the 2R-WGD^35–37^, and that the fourth member of the initial gene quartet was lost before the diversification of jawed vertebrates. If this is the case, we would expect to find conserved synteny between chromosomal regions containing *Bmp2*, -*4*, and -*16* genes in extant vertebrates. To test this hypothesis, we analysed the three-spined stickleback genome, as it does not retain any additional *bmp2/4/16* duplicates derived from the teleost-specific genome duplication (TSGD), which facilitates the identification of one-to-one correspondences in comparisons of gene arrays. We found eight pairs and three triplets of paralogs shared between the genomic regions containing *bmp2b*, -*4* and -*16* (Fig. 2). This gene-by-gene paralogy between the three 10 Mb-long chromosomal regions indicates their origin in a large-scale duplication. By investigating the timing of duplications of the neighbouring gene families (Fig. S1), we found that these genomic regions multiplied in the pre-vertebrate or early-vertebrate lineage after the split of the cephalochordate and tunicate lineages, and before the radiation of jawed vertebrates. The variable positioning of cyclostome genes in these trees does not allow us to infer the exact timing of the duplication event of this genomic region in relation to the divergence of cyclostomes from jawed vertebrates. This supports our hypothesis that the large-scale duplications giving rise to *bmp2b*, -*4* and -*16* containing chromosomal regions coincided with the 2R-WGD occurring early in vertebrate evolution^38^.

### Characterization of the *Bmp16* orthology group

**Figure 3:**
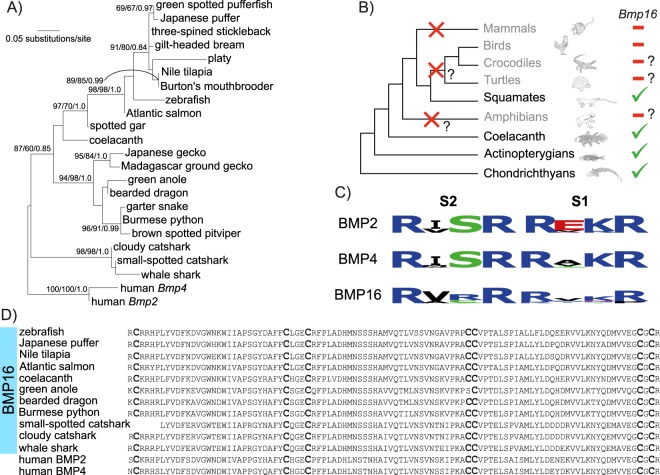
Phylogenetic tree of jawed vertebrate *Bmp16* genes and alignment of their deduced amino acid sequences. (**A**) Tree inference is based on the Dayhoff + Γ_4_ (shape parameter of gamma distribution α = 0.50) and an alignment of 96 amino acids. Support values at nodes are shown in order, bootstrap probabilities in the ML analysis and in the NJ analysis, and posterior probabilities in the Bayesian tree inference. Only bootstrap values no less than 70 in the ML analysis are shown. See Table S1 for accession IDs of included sequences. (**B**) Presences and presumed absences of *Bmp16* genes in major vertebrate lineages. Inferred secondary gene losses are indicated in the species tree with red crosses. Question marks indicate uncertainty about absence of *Bmp16* genes due to limited sequence resources. The phylogenetic position of turtles is based on existing literature^44,47–49,77^. (**C**) Conservation of the two proteolytic cleavage sites S1 and S2. The minimal motif Arg-X-X-Arg (R-X-X-R) is conserved in all BMP2/4/16 proteins, except for a few BMP16 proteins. The conservation level of the cleavage sites was visualized using the software WebLogo (URL: http://weblogo.berkeley.edu^71^). (**D**) Alignment of the C-terminus of diverse BMP16 and human BMP2 and -4 proteins. Cysteine residues (‘C’) that are involved in the formation of the cysteine-knot motif are shown in bold. This amino acid site is conserved throughout the alignment, except for *A*. *carolinensis* BMP16 whose fourth cysteine is substituted by a serine residue (‘S’).

The phylogenetic analysis focusing on *Bmp16* genes resulted in a gene tree that recovered monophyly for the following individual taxa: chondrichthyans, squamates, and teleosts (99/98, 98/98, and 91/98, respectively; Fig. 3A). The *Bmp16* gene tree reflects the expected phylogenetic relationships between species^39–42^, with the exceptions of the position of the Atlantic salmon *bmp16* gene that should be more closely related to other teleost *bmp16* genes relative to the zebrafish ortholog, the position of the small-spotted catshark that should be sister to the cloudy catshark instead of the whale shark, and the African coelacanth should show higher affinities to squamates than to actinopterygians (Fig. 3A). By mapping the identified *Bmp16* genes onto the vertebrate species phylogeny, we inferred the presumed absences of *Bmp16* genes from some vertebrate lineages (Fig. 3B) in accordance with previously published results^3^. This revealed putative secondary gene losses in mammals, amphibians, and archelosaurs^43,44^ (birds, crocodiles, and turtles; Fig. 3B). The absence of *Bmp16* orthologs in the thornback ray and the elephant fish putatively suggests two additional and independent losses of *Bmp16* in the Batoidea (rays, skates, and torpedoes) and Holocephali (chimaeras) lineages, a finding that should be confirmed with genome-wide information of more species in individual taxa in the future.

To assess if BMP16 proteins retain structural characteristics typical of related BMP proteins, we investigated the conservation of functionally described amino acid residues. The described motif Arg-X-X-Arg (R-X-X-R)^45^ of proteolytic cleavage sites S1 and S2^23^ is largely conserved in deduced amino acid sequences of jawed vertebrate BMP2/4/16 proteins (Fig. 3C). However, the level of conservation is lower in BMP16 proteins (average entropy of S1 and S2 of BMP2, 0.21; of BMP4, 0.26; of BMP16, 0.63), in particular in the S1 motif, possibly indicating a lower predisposition for cleavage at this motif (Fig. 3C). We examined an amino acid alignment containing representative BMP16 proteins for the seven cysteine residues essential for cysteine-knot formation and find that they are generally conserved in BMP16 proteins (Fig. 3D), with the only exception of the green anole BMP16 protein in which the fourth cysteine residue is substituted by a serine residue (Fig. 3D). Taken together, BMP16 proteins that are retained by chondrichthyans, squamates, actinopterygians and the coelacanth contain structural characteristics of functional BMP proteins.

### Gene expression analysis of *Bmp2/4/16* genes in catshark, zebrafish and green anole

Descriptions of *Bmp16* gene expression patterns have hitherto been restricted to teleost fishes^2,3,5^. We used RNA-seq as well as *in situ* hybridisation to gain information on embryonic gene expression profiles in chondrichthyans and squamates to infer functional diversifications within the *Bmp2/4/16* group of genes.

**Figure 4:**
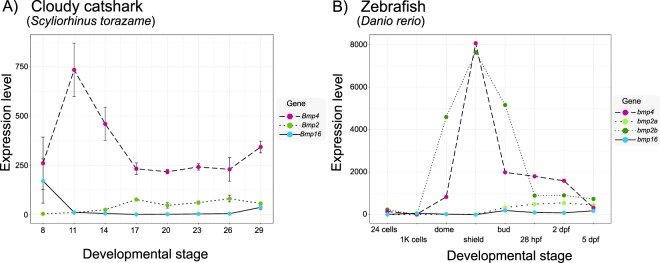
Ontogenetic gene expression levels of *Bmp2*, *-4* and -*16* in the cloudy catshark and the zebrafish. Plots show a developmental time course of gene expression levels (in FPKM values) of (**A**) cloudy catshark *Bmp2*, -*4* and -*16* genes (with standard errors derived from three biological replicates) and (**B**) zebrafish *bmp2a*, -*2b*, -*4* and -*16* genes. Sequencing data for the cloudy catshark was obtained in this study, while values for zebrafish were extracted from the literature (without replicates)^46^. Note the expression levels between the two species should not be compared since FPKM values are strongly influenced by the manners of sequence data acquisition, read trimming, and read mapping. Abbreviations: 1 K, 1000; hpf, hours post fertilization; dpf, days post fertilization.

We analysed RNA-seq data sampled in a developmental series spanning eight time points (from stage 8 to 29) of the cloudy catshark, and analysed pre-existing transcriptome data for a developmental series of eight stages (from 24 cells to 5 days post fertilization) of the zebrafish^46^ (Fig. 4). Within-species comparison of *Bmp2* (*a* and *b*), -*4* and -*16* expression levels (FPKM values, see Methods) revealed that *Bmp4* of the cloudy catshark (Fig. 4A), as well as *bmp4* and *bmp2b* of the zebrafish (Fig. 4B), have strikingly higher expression levels than their paralogs. Over the course of development, expression levels of these three genes are peaking at early/mid-developmental stages, roughly corresponding to gastrulation (stage 11 in the cloudy catshark and shield stage in zebrafish; Fig. 4).

**Figure 5:**
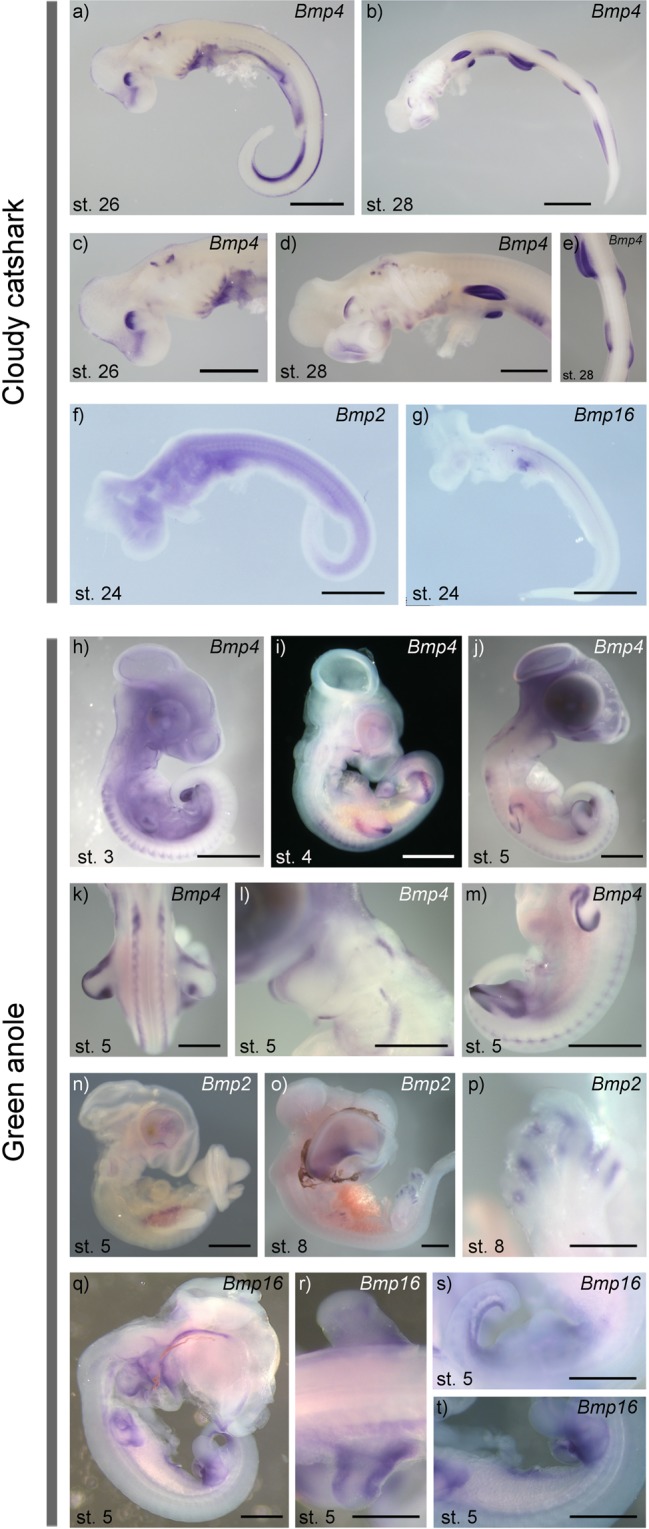
Gene expression patterns of *Bmp2*, -*4* and -*16* genes in the cloudy catshark and the green anole lizard. *In situ* hybridisation in the cloudy catshark showed that *Bmp4* is expressed in the dorsal part of the retina (**a**–**d**) the olfactory epithelium (**a**–**d**) the otic vesicle (**a**–**d**) the median fin fold (**a**) the ventral part of the branchial arches (**a**,**c**,**d**), the heart (**a**,**c**), the tail bud mesenchyme (**a**) and the paired fin buds (**b**,**d**,**e**). At stage 24, catshark *Bmp2* is diffusely expressed in mesodermal tissue (**f**) and *Bmp16* shows expression signal in the heart and notochord (**g**). In the green anole, *Bmp4* is expressed in the dorsal part of the retina (**h**,**i**), dorsal root ganglia (h,k,m) the ventral part of the branchial arches (**j**,**l**), the otic vesicle (**l**) and the limb buds (**i**–**k**,**m**). *Bmp2* expression in the green anole was not evident until stage 5 at which it was expressed in the gut-associated mesoderm (**n**) and stage 8 at which it was expressed in the dorsal part of the retina (**o**) and the interdigital tissue (**o**,**p**). *Bmp16* in the green anole is comparatively widely expressed at stage 5 with expression domains in the dorsal retina (**q**) the heart (**q**) limb buds (**r**) ventral tail tissue (**s**) and gut-associated mesoderm. (**s**,**t**) Scale bar: 2 mm.

*In situ* hybridisation revealed that *Bmp4* is widely expressed in both the cloudy catshark at stages 26 and 28 as well as the green anole lizard at stages 3, 4, and 5 (Fig. 5). In the catshark, *Bmp4* is expressed in the dorsal part of the retina, the olfactory epithelium, the otic vesicle, the median fin fold, the ventral part of the branchial arches, and the paired fin buds (Fig. 5a–g). In the lizard, *Bmp4* is expressed in the dorsal part of the retina, the otic vesicle, dorsal root ganglia, the ventral part of the branchial arches, and the limb buds (Fig. 5h–m). *Bmp2* is diffusely expressed in mesodermal tissue (Fig. 5f) at stage 24 in the cloudy catshark, while *Bmp2* expression in the green anole was not evident until stage 5 at which it was expressed in the gut-associated mesoderm (Fig. 5n,o), and stage 8 at which it was expressed in the dorsal part of the retina and the interdigital tissue (Fig. 5o,p). *Bmp16* shows expression signals in the heart and notochord at stage 24 in the cloudy catshark (Fig. 5g). *Bmp16* in the green anole is comparatively widely expressed at stage 5 with expression domains in the dorsal retina, the heart, ventral tail tissue, limb buds and gut-associated mesoderm (Fig. 5q–t). In summary, *Bmp4* is broadly expressed in both embryonic catshark and green anole with several distinct domains (Figs 4 and 5). In contrast, *Bmp2* shows only limited developmental expression in both species, and *Bmp16* is broadly expressed in green anole, but not in catshark embryos (Figs 4 and 5).

## Discussion

Identification of *Bmp16* genes was until now confined to actinopterygian fishes, the green anole and the African coelacanth^2–5^. Through targeted sequencing efforts and database mining we identified *Bmp16* genes in chondrichthyans (two catshark species and the whale shark) and squamates (an agamid lizard, two geckos, and three snakes; Figs 1 and 3). This result refines the inventory of the *Bmp16* gene repertoire across the vertebrate tree of life and taxonomically narrows down the instances of secondary gene losses. A previous study claimed four independent gene loss events in the lineages leading to mammals, turtles, archosaurs (crocodiles and birds) and amphibians^3^. This inference was based on a hypothetical phylogenetic relationship of turtles branching before the split between lepidosaurs and archosaurs. However, in the widely accepted phylogenetic tree of sauropsids, turtle are positioned as sister taxon to archosaurs^43,44,47–49^, which reduces the estimated *Bmp16* gene losses identified by Marques *et al*.^3^ from four to three. Our survey based on enriched sequence resources confirmed that the *Bmp16* genes were likely lost three times during vertebrate evolution, namely at the base of mammals, archelosaurs (archosaurs and turtles), and amphibians (Fig. 3B). There could also have been additional *Bmp16* ortholog losses in the Batoidea and Holocephali lineages since we did not identify *Bmp16* in either the thornback ray or the elephant fish. In-depth taxonomic exploration of more amphibian, turtle, crocodile, or chondrichthyan genomes in the future might reveal a wider taxonomic distribution of *Bmp16* genes. This, however, seems unlikely for mammals and birds since a large number of genomes (71 mammalian genomes in the Ensembl database alone, and at least 44 bird genomes in Avianbase^50^, last accessed March 2018) has been searched for *Bmp16* genes, but none were detected. One factor likely contributing to the propensity of *Bmp16* to become lost is the functional redundancy between *Bmp16*, and *Bmp2* and -*4* genes in terms of activating BMP-signalling^3^.

Two previous studies addressed the question about the origin of the *Bmp2/4/16* group of genes in a phylogenetic framework^2,3^. Although molecular phylogenetics alone does not provide significant support for the exact timing of the *Bmp2/4/16* diversification, our extended dataset including a broader selection of *Bmp16* genes of elasmobranchs and three additional cyclostome *Bmp2/4/16* genes (*Bmp2/4/16-A*, *-B*, and *-C* of the inshore hagfish) provides a more robust basis for re-addressing this question. However, a gene family tree alone cannot resolve the question of the scale of the duplication giving rise to paralogous genes (single gene or chromosome/genome wide duplication). Genome-wide synteny analyses were shown to be an adequate tool to detect traces of whole genome duplications^51–53^. Our synteny analyses suggest the origin of *Bmp2*, -*4* and -*16* in a large-scale duplication event. By inspecting duplication patterns of neighbouring gene families (Fig. S1), we conclude that the duplication event creating the chromosomal triplet (Fig. 2) occurred after the split of tunicates, but before the radiation of jawed vertebrates, thus coinciding with the 2R-WGD at the dawn of vertebrate evolution^54^. This timing is supported by our reconstruction of the molecular phylogeny (Fig. 1), suggesting that *Bmp2*, -*4* and -*16* genes are likely remnants of a gene quartet originating through the 2R-WGD. As expected after two genome duplications within a short time frame, the relationships among gnathostome *Bmp2*, -*4* and -*16* genes remain controversial: our study finds weak support for a sister-group relationship of gnathostome *Bmp2* and -*16* genes (Fig. 1), while previous studies show mixed support for this scenario^2,3^. In addition, the relationship between gnathostome and cyclostome *Bmp2/4/16* genes cannot be unambiguously inferred. While our ML and Bayesian analyses suggest that the triplication of the ancestral gnathostome *Bmp2/4/16* gene happened after the split from cyclostomes, we cannot rule out a possible orthology relationship between e.g. cyclostome *Bmp2/4/16* genes and gnathostome *Bmp16* genes, as suggested by previous studies^2,3^. Despite that we were not able to resolve the question about the relationships between cyclostome and gnathostome Bmp2/4/16 homologs, our analyses (Figs. 1, 2 and S1) solidly support the origin of jawed vertebrate *Bmp2*, -*4* and -*16* genes in a triplication event that was completed before the chondrichthyan/osteichthyan divergence.

A striking feature of the phylogenetic tree of the *Bmp2/4/16* subgroups is the increased branch length of the *Bmp16* group of genes compared to those of *Bmp2* and -*4*. Our estimation of evolutionary rates between teleost *bmp2*, -*4* and -*16* gene pairs revealed that this is not caused by an increased mutation rate of the *bmp16* locus, as described, for example, for the teleost *HoxA13a* gene^55^. The differences in evolutionary rate of *Bmp16* instead appears to be caused by higher purifying selection pressures on *Bmp2* and -*4* compared to *Bmp16* genes as evidenced by elevated ω_groups_ values in the latter (Fig. 1).

**Figure 6:**
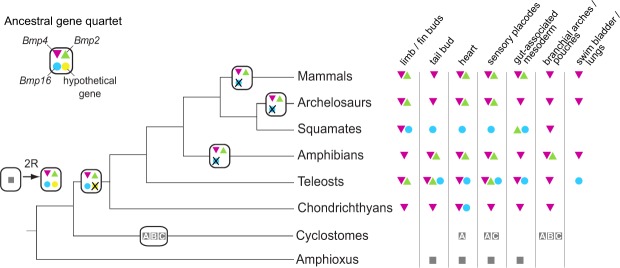
Evolutionary scenario of the *Bmp2/4/16* diversification in vertebrates. Quadruplication of the ancestral *Bmp2/4/16* gene was followed by lineage-specific losses of *Bmp16* in mammals, archelosaurs and amphibians. Orthology relationships between cyclostome and gnathostome *Bmp2*, -*4* and -*16* genes could not be definitely resolved by the present study. Therefore, this scenario is not assuming any orthology relationships between gnathostome and cyclostome *Bmp2/4/16* genes. Gene symbols in the matrix on the right side indicate evidence of expression in certain tissues during embryonic development. Information on expression domains were collected from the literature^8–13,15,20,56,78–86^ as well as from this study. Absence of gene expressions might in some cases, e.g. squamates and chondrichthyans, be attributed to non-exhaustive gene expression analyses rather than actual loss of expression domains. Note that the expression domain ‘limb/fin bud’ only refers to the early stages of limb/fin bud development and excludes apoptotic Bmp-signalling at later stages in interdigital tissue.

Our expression profiling of *Bmp4* genes in the cloudy catshark and the green anole lizard revealed conserved expression patterns in accordance with previous reports in other vertebrates^8–13^. Because of limited availability of embryonic stages, our survey of *Bmp2*, *-4* and -*16* expression patterns is not complete. Therefore, we cannot address possible losses of expression domains in reptiles and chondrichthyans. The high level of gene expression conservation of *Bmp4* throughout jawed vertebrates is in stark contrast to major differences in *Bmp2* and -*16* expression patterns between the zebrafish^2^, the cloudy catshark and the green anole (Fig. 6). Although comparison of expression patterns between species with different body plans and developmental dynamics is not straightforward, we observed marked differences. Using *Bmp2/4/16* expression profiles described in the literature complemented by expression patterns in the cloudy catshark and the green anole lizard that were collected in this study, we were able to reconstruct shuffling of expression domains across vertebrate (Fig. 6). By comparing expression profiles of jawed vertebrate *Bmp2/4/16* genes with those of their amphioxus ortholog (*AmphiBMP2/4*)^56^, one can infer that the pre-duplication *Bmp2/4/16* gene was likely expressed in the tail bud, heart, sensory placodes and gut-associated mesoderm of a vertebrate ancestor (Fig. 6). Developing limbs and swim bladder (or lungs) are vertebrate novelties without homologous structures in amphioxus that newly co-opted Bmp2/4/16 signalling in their underlying developmental pathways (Fig. 6). This inferred scenario also illustrates that *Bmp4* orthologs in jawed vertebrates are most broadly expressed, and presumably kept the expression domains of the ancestral *Bmp2/4/16* gene with only very few losses of expression domains (e.g., *Bmp4* expression has not been reported for the squamate heart). Through subfunctionalization, the *Bmp16* gene has presumably kept ancestral expression domains that were lost by other *Bmp2/4* genes (e.g., zebrafish swim bladder), but also retained expression domains redundant with other *Bmp2/4* genes (e.g., green anole limb buds and zebrafish sensory placodes; Fig. 6). Our non-exhaustive investigation of (temporal) expression profiles (Figs 4 and 5) and putatively incomplete descriptions in existing literature preclude a more fine-scale and taxon-dense analysis of losses and gains of expression domains. In summary, our evolutionary scenario illustrates that expression domains of vertebrate *Bmp2/4/16* genes were frequently reshuffled during vertebrate evolution with *Bmp16* genes showing the least conserved expression profiles (Fig. 6).

## Conclusion

Taken together, our study provides a rich description of divergent evolutionary fates after whole genome duplication on the example of the *Bmp2/4/16* group of genes. We describe asymmetric patterns of evolution between *Bmp16* and its sister genes *Bmp2* and -*4* in terms of molecular sequence evolution, propensity for gene loss and diversification of expression profiles in development. While the prevalence of asymmetric divergence of gene duplicates has been previously recognized^57,58^, case studies integrating insights from diverse aspects of gene family evolution are crucial for evaluating the extent to which different characteristics are correlated. Our study indicates that propensity for gene loss and rate of sequence evolution are tightly correlated with fast-evolving genes being more likely to get lost. In contrast, propensity for gene loss and rate of sequence evolution seem to be largely uncoupled from the shuffling of expression profiles in development: the fast-evolving, loss-prone *Bmp16* gene has largely retained ancestral expression domains in teleosts and squamates, but degenerated such patterns in chondrichthyans. Together with recent genome-wide approaches^59^, the present case study of the *Bmp16* gene provides us with clues about why some genes are lost and what characterizes loss-prone genes. Studies that combine insights from multiple aspects of gene family evolution have a role to play in furthering our understanding of the dynamics shaping gene repertoires on evolutionary time scales.

## Methods

### Animals

Shark and ray eggs were harvested in the Aquarium Facility of RIKEN Center for Developmental Biology (cloudy catshark, *S*. *torazame*), and the Sea Life Centre Konstanz (small-spotted catshark, *S*. *canicula*, and thornback ray, *R*. *clavata*), respectively. Eggs were kept in separate containers at 18 °C in oxygenated water until they reached the required stages^60^. Eggs of the green anole *A*. *carolinensis* were collected from in-house captive breeding colonies and incubated at 28 °C and ~70% humidity until they reached the required stages^61^. Embryos were dissected in cold DEPC-PBS and either fixed in 4% paraformaldehyde (PFA) for *in situ* hybridisation, or stored at −80 °C for RNA extraction. Our collaborators kindly provided sacrificed embryos of the Australian lungfish *N*. *forsteri* (for details see^62^), the Madagascar ground gecko *P*. *picta* and the Senegal bichir *P*. *senegalus*, cDNA of the cichlid *A*. *burtoni*, and RNA of a hybrid sturgeon embryos (*H*. *dauricus* female × *A*. *ruthenus* male) and brain tissue of an adult inshore hagfish (*E*. *burgeri*). Muscle tissue of an adult Florida gar (*L*. *platyrhinchus*) was obtained from a captive specimen.

### RT-PCR

Total RNA was extracted from the muscle tissue of *L*. *platyrhinchus* and embryos of *R*. *clavata*, *S*. *canicula*, *S*. *torazame*, *P*. *senegalus*, *N*. *forsteri*, *P*. *picta* and *A*. *carolinensis* by using TRIzol (Invitrogen). These isolated RNAs and RNAs of the hybrid sturgeon and *E*. *burgeri* were reverse transcribed into cDNA using SuperScript III (Invitrogen), following the instructions of 3′-RACE System (Invitrogen). All cDNAs were used as templates for degenerate PCRs using oligonucleotide primers that were designed based on amino acid residues shared either only among BMP2 and -4 proteins of diverse vertebrates, or only among BMP16 proteins. 3′-RACE PCRs were performed for first identifications of genes and 5′-RACE PCRs, the GeneRacer Kit (Invitrogen) or the SMARTer RACE Kit (Clontech) were used to obtain 5′-ends of cDNAs (for details of PCRs and primer sequences, see Tables S3 and S4).

### Retrieval of sequences

Sequences belonging to the *Bmp2/4/16* subgroup of genes were retrieved from the Ensembl genome database^63^ (version 69; URL: http://www.ensembl.org/) and NCBI Nucleotide database. We performed tblastn searches using human BMP2 and -4, and coelacanth BMP16 amino acid sequences as queries. In addition, we also performed targeted blast searches against project-based sequence databases of lineages with putative secondary losses of Bmp16 genes, i.e. amphibians and birds. We used the coelacanth BMP16 amino acid sequence as query against tblastn searches in the genome of *Ambystoma mexicanum*^64^ (assembly v3.0 and v4.0, URL: http://www.ambystoma.org/) and a database containing 44 bird genomes^50^ (URL: http://avianbase.narf.ac.uk/).

### Molecular phylogenetic analysis

An optimal multiple alignment of all retrieved DNA sequences (translated into amino acid sequences) was constructed using MEGA7^65^, in which the MUSCLE program^66^ is implemented. The best-fitting amino acid substitution models were estimated using ModelFinder^67^ implemented in the IQ-TREE software version 1.6.5^68^. Molecular phylogenetic trees were inferred using the regions that were unambiguously aligned with no gaps. Maximum-likelihood (ML) trees were inferred using IQ-TREE^68^, while neighbour-joining (NJ) trees were inferred using MEGA7. Bayesian tree inference was conducted using PhyloBayes version 4.1^69^ implementing an ‘automatic stopping rule’ (threshold for maximum difference, 0.1 and for effect size, 100). The phylogenetic analysis shown in Fig. 1 was conducted based on deduced amino acid sequences of selected *Bmp2*, -*4* and -*16* genes and invertebrate chordates as outgroup. This dataset excluded several truncated sequences identified in our degenerate PCR screens (Table S2). To estimate sequence conservation of the S1 and S2 cleavage sites, we extracted amino acids for both motifs for BMP2, -4 and -16 proteins and estimated their entropies for each of the six groups in BioEdit^70^ version 7.2.6, and used them as input for the software WebLogo (URL: http://weblogo.berkeley.edu)^71^. The second phylogenetic tree focusing on *Bmp16* genes (Fig. 3A) included all identified *Bmp16* genes except for the partial *Poeciliopsis turneri* ortholog (see Tables S1 and S2 for accession IDs of sequences). Both amino acid alignments are accessible on FigShare (DOI: 10.6084/m9.figshare.6938333 and 10.6084/m9.figshare.6938348).

### Tests of evolutionary rates

We estimated substitution rates for orthology groups of *Bmp2*, -*4* and -*16* genes using the same dataset as used in the phylogenetic reconstruction shown in Fig. 1, but with an alignment of codons instead of amino acids (822 base pairs; accessible on FigShare; DOI: 10.6084/m9.figshare.6940091). The codon alignment was obtained by back-translating the amino acid alignment into nucleotides using MEGA7. For the estimation of relative rates, we used the software package RRTree^72^ and calculated the number of non-synonymous substitutions (*K*_*a_groups*_) and transversions (*B*_*a*_) per non-synonymous site for groups of gnathostome *Bmp2*, -*4* and -*16* genes. The software also provides pairwise comparisons of *K*_*a_groups*_ and *B*_*a*_ values between the groups and *P* values for the likelihood that differences in rates are due to chance. The computation of the number of synonymous substitutions (*K*_s_groups_) and transitions (*A*_s_) per synonymous site failed, likely due to saturation of synonymous substitutions. We used codeml in the software package PAML version 4.9^73^ for maximum-likelihood estimations of the numbers of nonsynonymous substitutions per nonsynonymous site (*K*_*a_groups*_) and the number of synonymous substitutions per synonymous sites (*K*_*s_groups*_), as well as their ratios (ω_groups_ = *K*_*a*_/*K*_*s*_). Since our main interest here was to compare evolutionary rates between the three vertebrate Bmp orthology groups, we constrained rates to be equal within each group. The observed numbers of synonymous and non-synonymous substitutions per gnathostome Bmp orthology group are given in Table S5. In addition, we estimated pairwise *K*_*a_pairwise*_/*K*_*s_pairwise*_ and ω_pairwise_ values for *Bmp2*, -*4* and -*16* genes of species pairs using the program yn00 in the package PAML version 4.9.

### Synteny analyses

We searched for conserved synteny between the genomic regions of *Bmp2*, -*4* and -*16* to test the hypothesis that these duplicates are derived from a large-scale duplication event^74^. Using the Ensembl BioMart interface, we downloaded a set of paralogous genes shared between 10 Mb chromosomal regions flanking *bmp2b*, -*4* and -*16* in the three-spined stickleback genome. This set of paralogs was filtered using Ensembl ‘Gene Tree’, and we retained only pairs, triplets of quartets of paralogs whose duplication pattern is in accordance with the 2R-WGD. We constructed phylogenetic trees for these gene families to confirm their evolutionary origin (Fig. S1). The locations of these paralogs were plotted onto the corresponding three-spined stickleback chromosomes (i.e., ‘linkage groups’; Fig. 2).

### Expression quantification using RNA-seq

We used FASTQ files (DDBJ DRA ID DRR111753-DRR111773) that contained RNA-seq data derived from cloudy catshark embryos at stages 8, 11, 14, 17, 20, 23, and 26 in three biological replicates. Adapter sequences and low-quality bases (<Q30) were trimmed from the 3′-ends by trim_galore (http://www.bioinformatics.babraham.ac.uk/projects/trim_galore/), in which cutadapt is implemented^33^, and reads shorter than 50 bp after adapter and quality trimming were discarded. Low-quality reads in which proportion of the bases ≥Q30 was less than 80% were discarded by the program fastq_quality_filter in FASTX Toolkit 0.0.13 (http://hannonlab.cshl.edu/fastx_toolkit/index.html). After quality control, reads were mapped onto the transcript sequences including the full-length ORF and untranslated regions of *S*. *torazame Bmp2*, -*4* and -*16*, with the program eXpress version 1.5.1. Gene expression levels were expressed as fragments per kilobase of exon model per million mapped reads (FPKM).

### *In situ* hybridisation of *Bmp2*, -*4 and* -*16* genes

Riboprobes used in *in situ* hybridisations were produced based on 3′- and 5′-fragments of *S*. *torazame Bmp2*, -*4*, and -*16*, and *A*. *carolinensis Bmp16* cDNAs (for information of cDNA preparation, see Table S3; for information on primer sequences, see Table S6). Whole-mount *in situ* hybridisations were performed according to a protocol that was originally developed for snake and lizard embryos (Nicolas Di-Poϊ, personal communication) for green anole embryos, and based on O’Neill *et al*.^75^ for *S*. *torazame* embryos. Riboprobes were labelled with digoxigenin-UTP (Roche Applied Science) and hybridisation was detected with alkaline phosphate-conjugated anti-digoxigenin antibody followed by incubation with nitroblue tetrazolium and BCIP (5-bromo-4-chloro-3-indolyl phosphate). Stained embryos were examined with a Zeiss Axiophot microscope. Images were processed using Zeiss Axiovision and Adobe Photoshop software.

## Supplementary information

Suplementary Information

Supplementary file 1

## Data Availability

All newly identified sequences are deposited in EMBL under accession numbers (study accession number, PRJEB25510; accession IDs, LT989953- LT989973). All alignments are accessible on FigShare (DOI: 10.6084/m9.figshare.6938372, 10.6084/m9.figshare.6938369, 10.6084/m9.figshare.6938366, 10.6084/m9.figshare.6938363, 10.6084/m9.figshare.6938360, 10.6084/m9.figshare.6938357, 10.6084/m9.figshare.6938354, 10.6084/m9.figshare.6938333, 10.6084/m9.figshare.6938348 and 10.6084/m9.figshare.6940091).
