# Supplementary material for: Asymmetric paralog evolution between the “cryptic” gene Bmp16 and its well-studied sister genes Bmp2 and Bmp4
Source: Sci Rep. 2019 Feb 28;9:3136. doi: 10.1038/s41598-019-40055-1 (PMC6395752; doi:10.1038/s41598-019-40055-1)
Supplement: Supplementary file 1 — Suplementary Information [file 41598_2019_40055_MOESM1_ESM.pdf]

Supplementary material

Asymmetric paralog evolution between the "cryptic" gene *Bmp16* and its well-studied sister genes *Bmp2* and *Bmp4*

Nathalie Feiner, Fumio Motone, Axel Meyer and Shigehiro Kuraku

Supplementary figure

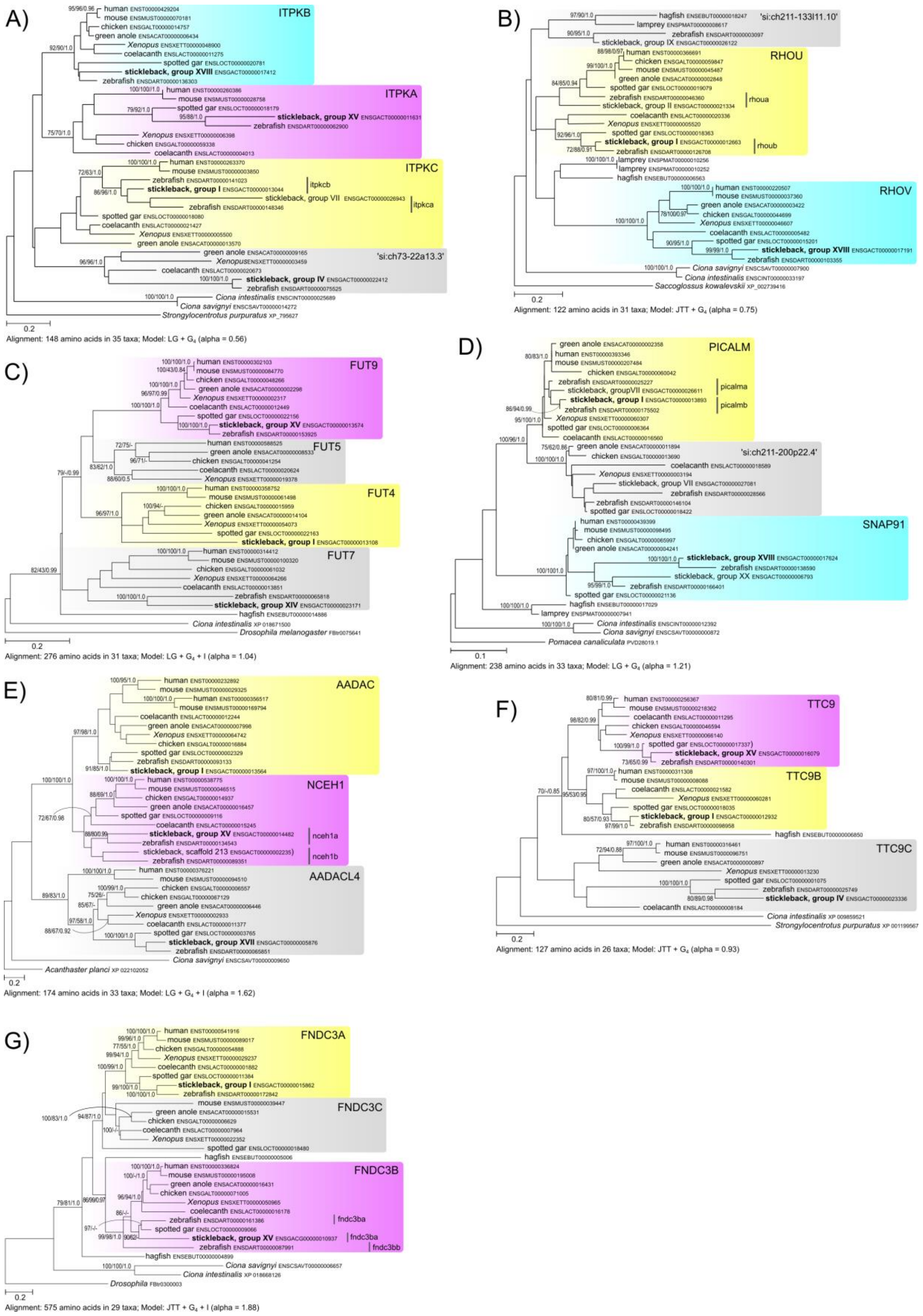

Figure S1. Phylogenetic trees of gene families whose members surround the *Bmp2, -4* and *-16* genes. Panels A-G show ML trees (inferred with MEGA7[1]) of pairs or triplets of genes indicated in the synteny blocks shown in Fig. 2. Information on tree inference parameters are given below each tree. Scale bar shows the number of substitutions per site. Colored backgrounds highlight orthology groups of gnathostome genes: yellow for stickleback genes on group I, blue for group XVIII, purple for XV, and grey for stickleback genes in other genomic regions. Names for each orthology group are based on human nomenclature. Support values at nodes are shown in order, bootstrap probabilities in the ML method and in the NJ method, and posterior probabilities in the Bayesian tree inference. Only bootstrap values no less than 70 in the ML analysis are shown. All amino acid alignments are accessible on FigShare (DOI: 10.6084/m9.figshare.6938372, 10.6084/m9.figshare.6938369, 10.6084/m9.figshare.6938366, 10.6084/m9.figshare.6938363, 10.6084/m9.figshare.6938360, 10.6084/m9.figshare.6938357 and 10.6084/m9.figshare.6938354).

Supplementary tables

Table S1. Sequences included in molecular phylogenetic analyses shown in Fig. 1 and Fig. 3A, and synteny analysis shown in Fig. 2.

Latin species name	Common name	Gene name	Accession ID	Included in Figure(s)
Ambystoma mexicanum	Mexican salamander	*Bmp2*	EU339232	Fig. 1
Anolis carolinensis	green anole	*Bmp2*	ENSACAT00000003102	Fig. 1
Anolis carolinensis	green anole	*Bmp4*	ENSACAT00000017970	Fig. 1
Anolis carolinensis	green anole	*Bmp16*	LT989953	Figs. 1, 3A
Astatotilapia burtoni	Burton's mouthbrooder	*bmp16*	LT989956	Fig. 3A
Branchiostoma floridae	Florida lancelet	*AmphiBMP2/4*	XM_002596812	Fig. 1
Callorhinchus milii	elephant fish	*Bmp2*	XM_007905506	Fig. 1
Callorhinchus milii	elephant fish	*Bmp4*	XM_007888150	Fig. 1
Ciona savignyi	Solitary sea squirt	*Bmp2/4*	ENSCSAVT00000004520	Fig. 1
Danio rerio	zebrafish	*bmp2a*	ENSDART00000019364	Fig. 1
Danio rerio	zebrafish	*bmp2b*	ENSDART00000131169	Fig. 1
Danio rerio	zebrafish	*bmp4*	ENSDART00000075150	Fig. 1
Danio rerio	zebrafish	*bmp16*	NM_001171776	Figs. 1, 3A
Eptatretus burgeri	inshore hagfish	*Bmp2/4/16-A*	see Supplementary File 1	Fig. 1
Eptatretus burgeri	inshore hagfish	*Bmp2/4/16-B*	see Supplementary File 1	Fig. 1
Eptatretus burgeri	inshore hagfish	*Bmp2/4/16-C*	see Supplementary File 1	Fig. 1
Gallus gallus	chicken	*BMP4*	ENSGALT00000037192	Fig. 1
Gasterosteus aculeatus	stickleback	*bmp2*	ENSGACT00000016539	Fig. 2
Gasterosteus aculeatus	stickleback	*bmp4*	ENSGACT00000013724	Fig.2
Gasterosteus aculeatus	stickleback	*bmp16*	ENSGACT00000012714	Figs. 2, 3A
Gekko japonicus	Japanese gecko	*Bmp2*	XM_015405598.1	Fig. 1
Gekko japonicus	Japanese gecko	*Bmp4*	XM_015420851.1	Fig. 1
Gekko japonicus	Japanese gecko	*Bmp16*	XM_015427264.1	Figs. 1, 3A
Homo sapiens	human	*BMP2*	ENST00000378827	Figs. 1, 3A
Homo sapiens	human	*BMP4*	ENST00000245451	Figs. 1, 3A
Huso dauricus ♀ x *Acipenser ruthenus* ♂	Bester	*Bmp2*	LT989959	Fig. 1
Huso dauricus ♀ x *Acipenser ruthenus* ♂	Bester	*Bmp4*	LT989960	Fig. 1
Latimeria chalumnae	African coelacanth	*Bmp2*	ENSLACT00000016280	Fig. 1
Latimeria chalumnae	African coelacanth	*Bmp4*	ENSLACT00000021864	Fig. 1
Latimeria chalumnae	African coelacanth	*Bmp16*	ENSLACT00000009133	Figs. 1, 3A
Lepisosteus oculatus	spotted gar	*Bmp2*	ENSLOCT00000020341	Fig. 1
Lepisosteus oculatus	spotted gar	*Bmp4*	ENSLOCT00000014951	Fig. 1
Lepisosteus oculatus	spotted gar	*Bmp16*	ENSLOCT00000018333	Figs. 1, 3A
Oreochromis niloticus	Nile tilapia	*bmp16*	ENSONIT00000007507	Fig. 3A
Oryzias latipes	medaka	*bmp2b*	ENSORLT00000012260	Fig. 1
Oryzias latipes	medaka	*bmp4*	ENSORLT00000016674	Fig. 1
Paroedura picta	Madagascar ground gecko	*Bmp16*	LT989964	Figs. 1, 3A
Petromyzon marinus	sea lamprey	*PmBMP24-A*	AY602220	Fig. 1
Petromyzon marinus	sea lamprey	*PmBMP24-B*	AY602221	Fig. 1
Petromyzon marinus	sea lamprey	*PmBMP24-C*	AY602222	Fig. 1

Pogona vitticeps	bearded dragon	*Bmp2*	XM_020793685.1	Fig. 1
Pogona vitticeps	bearded dragon	*Bmp16*	XM_020810881.1	Figs. 1, 3A
Polypterus senegalus	Senegal bichir	*Bmp4*	LT989966	Fig. 1
Protobothrops mucrosquamatus	brown-spotted pit viper	*Bmp2*	XM_015813549	Fig. 1
Protobothrops mucrosquamatus	brown-spotted pit viper	*Bmp4*	XM_015822447	Fig. 1
Protobothrops mucrosquamatus	brown-spotted pit viper	*Bmp16*	XM_015829826	Fig. 3A
Ptychodera flava	acorn worm	*Pf-BMP2/4*	AB028219	Fig. 1
Python bivittatus molurus	Burmese python	*Bmp2*	XM_007427029	Fig. 1
Python bivittatus molurus	Burmese python	*Bmp4*	XM_007436872	Fig. 1
Python bivittatus molurus	Burmese python	*Bmp16*	AEQU010318313, AEQU010313080	Fig. 3A
Raja clavata	thornback ray	*Bmp2*	LT989967	Fig. 1
Raja clavata	thornback ray	*Bmp4*	LT989968	Fig. 1
Rhincodon typus	whale shark	*Bmp2*	XP_020370331	Fig. 1
Rhincodon typus	whale shark	*Bmp4*	XP_020366079	Fig. 1
Rhincodon typus	whale shark	*Bmp16*	XP_020370388	Figs. 1, 3A
Salmo salar	Atlantic salmon	*bmp16*	CK879241	Fig. 3A
Scyliorhinus canicula	small-spotted catshark	*Bmp2*	LT989969	Fig. 1
Scyliorhinus canicula	small-spotted catshark	*Bmp4*	LT989970	Fig. 1
Scyliorhinus canicula	small-spotted catshark	*Bmp16*	LT989971	Fig. 3A
Scyliorhinus torazame	cloudy catshark	*Bmp2*	see Supplementary File 1	Fig. 1
Scyliorhinus torazame	cloudy catshark	*Bmp4*	see Supplementary File 1	Fig. 1
Scyliorhinus torazame	cloudy catshark	*Bmp16*	see Supplementary File 1	Figs. 1, 3A
Sparus aurata	gilt-head bream	*bmp16*	FM155149	Fig. 3A
Takifugu rubripes	Japanese puffer	*bmp16*	ENSTRUT00000029118	Fig. 3A
Tetraodon nigroviridis	green spotted pufferfish	*bmp2b*	ENSTNIT00000013839	Fig. 1
Tetraodon nigroviridis	green spotted pufferfish	*bmp4*	ENSTNIT00000020600	Fig. 1
Tetraodon nigroviridis	green spotted pufferfish	*bmp16*	ENSTNIT00000010748	Figs. 1, 3A
Thamnophis sirtalis	garter snake	*Bmp2*	XM_014068423	Fig. 1
Thamnophis sirtalis	garter snake	*Bmp4*	XM_014054780	Fig. 1
Thamnophis sirtalis	garter snake	*Bmp16*	XM_014055083	Figs. 1, 3A
Xenopus tropicalis	Western clawed frog	*Bmp2*	ENSXETT00000012124	Fig. 1
Xenopus tropicalis	Western clawed frog	*Bmp4*	ENSXETT00000018837	Fig. 1
Xiphophorus maculatus	platy	*bmp16*	ENSXMAT00000004285	Fig. 3A

Species names are shown in alphabetic order.

Table S2. Sequences identified in this study but excluded from phylogenetic analysis.

Latin species name	Common name	Gene name	Accession ID
Astatotilapia burtoni	Burton’s mouthbrooder	*bmp2*	LT989954
Astatotilapia burtoni	Burton’s mouthbrooder	*bmp4*	LT989955
Eptatretus burgeri	inshore hagfish	*Bmp2/4/16-A*	LT989957
Huso dauricus	Kaluga sturgeon	*Bmp2*	LT989958
Lepisosteus platyrhincus	Florida gar	*Bmp4*	LT989961
Neoceratodus forsteri	Australian lungfish	*Bmp4*	LT989965
Paroedura picta	Madagascar ground gecko	*Bmp2*	LT989962
Paroedura picta	Madagascar ground gecko	*Bmp4*	LT989963

Table S3. Information of sequences identified in this study by RT-PCR.

Species	Gene name	Source of tissue/stage	Primer set used for first identification (3'RACE)	Method used for sequence extension	Completeness of cDNA
Anolis carolinensis	*Bmp16*	stage 8.5	NF169/170	5'RACE	full-length
Astatotilapia burtoni	*bmp2*	14 / 21 dpf[c]	NF5/6	GeneRacer kit[f]	full-length
Astatotilapia burtoni	*bmp4*	14 / 21 dpf[c]	NF5/6	GeneRacer kit[f]	full-length
Astatotilapia burtoni	*bmp16*	14 / 21 dpf[c]	NF49/50	GeneRacer kit[f]	full-length
Eptatretus burgeri	*Bmp2/4-α*	adult brain tissue	NF5/6	5'RACE	full-length
Huso dauricus	*Bmp2*	[d]	SK101/103[a]	-	partial
Huso dauricus ♀ x *Acipenser ruthenus* ♂	*Bmp2*	stage E	NF17/18[a]	-	partial
Huso dauricus ♀ x *Acipenser ruthenus* ♂	*Bmp4*	stage E	NF5/NF8	-	partial
Lepisosteus platyrhincus	*Bmp4*	adult muscle tissue	NF5/6	-	partial
Neoceratudos forsteri	*Bmp4*	stages 30-40[e]	SK101/103[b]	-	partial
Polypterus senegalus	*Bmp4*	finfold phase of larvae	SK101/103[b]	-	partial
Raja clavata	*Bmp2*	stage 32	NF5/6		full-length
Raja clavata	*Bmp4*	stage 32	NF5/8		full-length
Scyliorhinus canicula	*Bmp2*	stage 33		GeneRacer kit[f]	full-length
Scyliorhinus canicula	*Bmp4*	stage 33		GeneRacer kit[f]	full-length
Scyliorhinus canicula	*Bmp16*	stage 33	NF71/72	5'RACE	partial
Scyliorhinus torazame	*Bmp2*	stage 23 / 25	NF5/6	-	partial
Scyliorhinus torazame	*Bmp4*	stage 23 / 25	NF5/6	-	partial
Scyliorhinus torazame	*Bmp16*	stage 23 / 25	NF71/72	5'RACE	partial
Paroedura picta	*Bmp2*	4 / 12 dpo	NF5/6	-	partial
Paroedura picta	*Bmp4*	4 / 12 dpo	NF5/6	-	partial

[a] Nested PCR was performed using the primer SK106 instead of an adapter primer.

[b] Nested PCR was performed using the primer SK105 instead of an adapter primer.

[c] see [2].

[d] see [3].

[e] see [4].

[f] purchased from Thermo Fisher Scientific

Table S4. Sequences of primers used in this study for identification of novel sequences.

Primers used in PCRs	Primer sequence (5’ → 3’)	Amino acid sequence
NF5 (F)	GACTTYWSNGAYGTNGGNTGGAA	DFSDVGWN
NF6 (F)	GGACCACCTNAAYWSNACNAAYCA	DHLNSTNH
NF8 (F)	AAGNGCNTGYTGYGTNCCNAC	P(R/K)ACCVPT
NF17 (F)	GCCAACACTGTYMGNNSNTTYCA	ANTVR(S/G)FH
NF18 (F)	ACCGTYMGYKSNTTYCAYCAYGA	TVR(G/S)FHH(D/E)
NF49 (F)	ATAGCWCCNWSNGGNTAYGAYGC	IAPSGYDA
NF50 (F)	CCGASNGGMTAYGAYGCNTTYTTY	PSGYDAFF
NF71 (F)	AACTCCTCCAGCCATGCCAT	NSSSHAI
NF72 (F)	ATCGTGCAGACGCTGGTCAA	IVQTLVN
SK101 (R)	GTCGTGCCNCCNTAYATGYTNGAYYT	VVPPYMLDL
SK103 (R)	GTGCCGCCNTAYATGYTNGAYYTNTA	VPPYMLDLY
SK105	TACANCCRCANCCYTCNACNACCAT	MVVEGCGCX
SK106	TCCRCANCCYTCNACNACCATNTC	DMVVEGCG

Table S5. Number of observed synonymous and non-synonymous substitutions per group of *Bmp* genes.

Group of genes	ω	Observed number of non-synonymous substitutions	Observed number of synonymous substitutions
gnathostome *Bmp2*	0.07	18.51	76.40
gnathostome *Bmp4*	0.05	13.62	80.54
gnathostome *Bmp16*	0.12	48.8	128.14

All values are given as average for the respective group of genes as provided by the software PAML version 4.9[5]. For more information, see Fig. 1.

Table S6. Sequences of primers used in cloning probes for *in situ* hybridization.

Gene	Species	Forward primer sequence (5’ → 3’)	Reverse primer sequence (5’ → 3’)	Length of probe
Bmp2	*A. carolinensis*	CCTCAACTCCACAAATCA	TTCTATTTGCTGAGCAGC	881 bp
Bmp4	*A. carolinensis*	TGATGGTCCTCCTATTGT	ACAATCCAATCGTTCCAG	992 bp
Bmp16	*A. carolinensis*	CCAAAATCAGTACCAAGTG	GATTGCTTTGATAGACTGG	760 bp
Bmp2	*S. torazame*	GGTTGTGGAAGGTTGTGG	AGGAGCTGCCCAGTGTTA	631 bp
Bmp4	*S. torazame*	ACCAGAGGAAGGGAGGAAAA	TTGTTCTTCTTGCGCCTCTG	764 bp
Bmp16	*S. torazame*	AGAGTACAGCACTGCAAC	CTCGTGGTTGGGAATGTT	611 bp

References

1. Kumar, S., Stecher, G. & Tamura, K. MEGA7: Molecular Evolutionary Genetics Analysis Version 7.0 for Bigger Datasets. *Molecular biology and evolution* **33**, 1870-1874 (2016).
2. Renz, A. J. *et al.* Ancestral and derived attributes of the dlx gene repertoire, cluster structure and expression patterns in an African cichlid fish. *EvoDevo* **2**, 1 (2011).
3. Fukamachi, S. & Meyer, A. Evolution of receptors for growth hormone and somatolactin in fish and land vertebrates: lessons from the lungfish and sturgeon orthologues. *J Mol Evol* **65**, 359-372 (2007).
4. Feiner, N., Ericsson, R., Meyer, A. & Kuraku, S. Revisiting the origin of the vertebrate Hox14 by including its relict sarcopterygian members. *Journal of experimental zoology. Part B, Molecular and developmental evolution* **316**, 515-525 (2011).
5. Yang, Z. PAML: a program package for phylogenetic analysis by maximum likelihood. *Comput Appl Biosci* **13**, 555-556 (1997).
