# Supplementary material for: Asymmetric paralog evolution between the “cryptic” gene Bmp16 and its well-studied sister genes Bmp2 and Bmp4
Source: Sci Rep. 2019 Feb 28;9:3136. doi: 10.1038/s41598-019-40055-1 (PMC6395752; doi:10.1038/s41598-019-40055-1)
Supplement: Supplementary file 2 — Supplementary file 1 [file 41598_2019_40055_MOESM2_ESM.pdf]

Asymmetric paralog evolution between the “cryptic” gene *Bmp16* and its well-studied sister genes *Bmp2* and *Bmp4*

Nathalie Feiner, Fumio Motone, Axel Meyer and Shigehiro Kuraku

This file contains three genes of the inshore hagfish *Eptatretus burgeri* and three genes of the cloudy catshark *Scyliorhinus torazame*.

The following three sequences have been identified in tblastn searches against the whole-genome shotgun contigs (wgs) of *Eptatretus burgeri* at NCBI (Assembly ID, GCA_900186335.2). Open reading frames have been manually curated. Below we provide the fasta header of the contig in the original genome assembly, followed by the curated mRNA sequence.

```
>Eptatretus burgeri Bmp2/4/16-A predicted coding sequence in
contig FYBX02009429.1
ATGATTCTCGGTAGCCTCTTGACGCTCATGACCCTGCTAGCGTCCCATCGCGTCTCGAGGGG
CGACATCGGCCTCGTCCCACGAGACGTGGCGGGGCTCCGGCTAGCACGGAGAGGCGATCCGG
CGCTGCCATCGCGGGCGGATGATGTGCAGCTCGCGGACTCTTTGCGCATCCTCGAACGGCGA
CTCCTCGACCTTTTTGGCCTCCAGCGACGGCCTCGGCCCTCCCCGAACGCCGTGGTGCCGGA
ATACATGTGGGAACTGTACAGAGGCGTCCGGCGGGTCCCGGCCGATCGCTCGCACGGCTCCT
CTGGGCTGTCCGTTCCGCGCGGTCTCTTCTCCAACACGGCCAGGAGCTTCCACCACGAAGAA
TTGCTCGAAGTCGTACCTCCGACGAGCTCGTCGTATCCCGGACGGCGCTACGTTTTCAACAT
GAGCAGCATACCCGAGAGAGAGGTCATTTCGGCAGCTGAACTGCGTCTATATCGCGCCCGAT
CATCCCCAGACCACCAGCGCCGGCAGAAGCAGCAGCGCATCAATGTGTACGAGGTCATGCGT
CCGGGTGCAGCGCCAGACGGAGTGTCCGTCGCTCGTCTGCTTGACACCCGCCTCGTGTGGAC
CAACGAGAGTGGGTGGGAGGCATTTGACGTTAGCCCGACGGTTTTGCGATGGACCGCAGCCA
CGGCTCACAACCATGGCATCTTGGTGGAGGTGTTGCAGGGCAATGACGAACTAACTCCCTGG
```

CGAGACGAAAGCCACAACGTGCGTCTAAGTCGGTCGCTGCACGACTACGAATCGGCCACCTG
GGCACAGCAGCGGCCACTGCTCGTCACCTTTGGTCACGATGGACGAAAGGATGCGACCCACG
TGGACCGGAGCGAAGAGGATGGTCGGACACGGAGCAAACGCCGTGCGGGCAAGCATCGATCA
GGAGGAGGTGGAAGAGGTGGGAGAGGTAAAGGAGGGGGTGGAGGAGGAGGACGCCGCAGTCG
GGGGACCTGCAACCGGCACCCTTTGTATGTCGATTTCAGTGATGTGGGCTGGAACGACTGGA
TCGTGGCTCCACCAGGCTACCACGCGTTTTTCTGCCAAGGTGAATGTCCCTTTCCCCTGGCC
GAGCACCTCAACTCCACCAACCACGCCATCGTGCAGACCCTCGTGAACTCGGTGAACCCGGC
GGTGCCCCGGGCATGCTGCGTCCCGACGGAACTGAGCGCCATCTCGCTACTTTACCTGGACG
AGTACGACAAGGTGGTACTGAAGAACTACCAAGACATGGTGGTGGAGGGTTGCGTTTGCCGC
TAA

>Eptatretus burgeri Bmp2/4/16-B predicted coding sequence in contig ScVlheP_9746
ATGACCATGCCGCCTACTCGAACCACTCTCATGGTGTTCGCGCTCTGCAGCACTCTGATCTA
CGATCTCGTCCACGGAACCATGCCGAGCTCGCACCTGGACACTGGTAGTTTACTGCCGCACC
TGGAAGCTCAACTTCTGGGGGTATTCGGTCTCAGGCGGCGGCCTCGGCCGCGAAGAGACGCT
GTGATCCCTCCCTACCTGCTTGAGATGTATCGATTGCACTCTGGGGAGCAACCTTCCGCAGG
AACCTTGAAGGACTTCGCCTTCTCGGAACGCGCGGCCTCTATTGCCAACACAGTTCGCGCTT
TCTATCATGAAGAATCGATTGAAGAACTAGATGTCTCGCCTGCAAATGCAGTATTTCGTGCC
ACGTTCAATCTAAGCAGCATACCTCCTGGGGAGGCTGTGCTGGCAGGTGAGCTGCGAATATT
CCGCGAAGGACATCGTGTGGCAGCTCCAGACTATAATGGACACCTCCGCATCAACGTATACG
AAAACCGGCAGATGGGGCGCGATCTAACCACTCGTCTATTGGACACACGTGTCCTCCATGTT
GGCTCCACCAGGTGGGAAAGTTTTGACGTGAGTCTGGCCTTGCTGAGGTGGACCCGTGTCCC
TGACTCCAACCTGGGACTTACGTTGGAGGTCACATCCCTCAACGGCACTTCTGATGGTATCC
TGCAAAGGACGTGGCACCACGTTCGCATCAGCCGCTCTGTGGGAGCACCACAACATAACCTA
GACACTCACTGGCCACTGCTGCGTCCGCTCCTGGTCACCTACAGCCACGATGGTAAAGGTCA
GGCTCTGAGAGGGTCGAGGCGTAAAAGGCGTGCAAGGCGCGGTAAGAAACATTTGAGGCGAC
TACGAGCGAACTGTCAGCGACATATGCTTTATGTGAACTTCACTGAGGTCAACTGGAATGAT
TGGATAGTTGCGCCACCCGGCTATCAAGCTTATTATTGCCAGGGCCAATGTCCTTTTCCACT
GGCTGAACACCTTAACTCCACAAACCATGCAATTGTGCAGACACTTGTGAACTCGGTGATTG
CGAGCATTCCACGTGCTTGCTGTGTCCCAACCACCCTGAGTCCCATCTCACTGTTGTATCTG
GATGAAGACGATAAGGTGGTTCTCAAAAACTATCAAGATATGGTGGTAGAAGGTTGTGGTTG
TCGATAG

>Eptatretus burgeri Bmp2/4/16-C predicted coding sequence in contig ScVlheP_10101
ATGTTGGCGAGCCCGCCATGCGCATTCGGCGCTCTGGATAACTCGGGCGAGGTGACCGGGTC
CAGCAGCTCAAACGAGCGGCTGTCGAAGGAGACCAGCTCGACCGCGCCACAGCTTTTGCCCA
CACTTGAGTCCCACCTCCTGGGAATGTTCGGGCTCGAGAAGAGGCCCCGGCCAGGGAGACGT
CCCGTTATCCCTCGATACCTGCTCGACCTCTACAGGCTGCAATCAGGAGAGGCTCCCTGGCC
GAGTTCCGAGTTGGAGTTGCCCGTGGGAGCGAGCCGAGCGAACACCGTGCGAGCATTTCACT
ACGACGAGTCATTGGATGAAAACGTTGACACATCATCATCATCATCATCATCATCATCATCT
GAGGTGCATCGGTTCATCTTCCCGCTGGACAGCGTCCCTGCAGGCGAGGAGGTCACGGCAGC
AGAGTTGAGGCTTTTCCGTTCACCACTTCACGACGCTGCTGACTCTGCTCGATGCTTCCACC
GTATCAACGTGTATGAGGTTTTACAGGGAAGAATGATGGAGGGAGTGGGATCGAGCACCATG
GGAGTGTGGATGGAGCAGTACGCGAGGGACAAAAATCGTCGGACAGAGCCCATTACTCGCTT
ACTGGACTCCAAACGCGTTCGTCATAACCAGACTCGTTGGGAAAGTTTTGACGTGAGTCCTG

CTGTGTTACGGTGGACAAGAGGTCAGGCACCAAACCTCGGTCTCCACGTTGAGATTGTGGCC
GAAGAAGGCTGCGAAGGAACCAATAGCACTGGGCATGTGCGGGTGAGCCGCTCGGTGTACAC
CAAGGACCAGGAGCGATGGTCACAGGTACAGCCACTGCTTGTTGCCTACAGCCATGATGGTG
GTGGCGCGACACTGATCCACAGATCCAAGAGGAGCCGAAAACGACCCAAAGGACGCAGGAGA
CACAAGCAGAACTGCCGGCGGCGCCACCTCTTCGCCAACTTTACCGAGCTCCGCTGGAACGA
TTGGATTGTTGCTCCACTGGGATACAACGCTTATTATTGTCAGGGTGAGTGTCCCTTCCCAT
TGGCAGAACATTTGAACTCCACGAATCATGCCATTGTACAAACTCTCGTGAACTCGGTGAAT
GAGAGCATCCCACGCGCTTGCTGTGTGCCCACCGAGCTGAGCGCAATCGCCATGCTCTACCT
GGATGAGTACAACAAAGTGGTGCTGAAGAACTACCAGGATATGGTGGTAGAAGGTTGCGGAT
GCCGCTAG

The following three sequences have been identified in assembled RNA-seq data of *Scyliorhinus torazame* embryos.

>*Scyliorhinus torazame Bmp2*
ATGACCGCGGGCAGACGACCTCTGATGGTGCTCCTGCTTTGTCAGGTCCTCTTCGGAGGCTC
GGCCGGGCTCATCCCCGAGATGGGCCGGAGGAAGTTCACCCAGCAGCAGGAGGCCAACTCGG
GGGGCCGGGCGAGCCCCCTGCAGACCGAGGGCATGCTCCAGGAGTTCGAGCTGCGGCTGCTC
AACATGTTCGGCCTGCGGCGCCGCCCTCAGCCCGGCAAGGAGCCGCTGATCCCGCACTACAT
GCTGGAGCTGTACCGGCTGCATTCGGAGGAGGAAGGGCAGCCTGGCACGCTCCTGCCCCGGT
TCCCCGAGAGGCCAGCCAGCCACGCCAACACAGTCAGGAGCTTCCACCACGAAGAACCAATG
GAGAAGCTGCCTGGAGCCAGAGGAGAGACAACCCGCCGATTTTTCTTCAGTTTGAACTCTAT
ACCGCATGAGGAGCTCATCACCTCGGCAGAATTGAGGATCTACCGCGAGCAGGAGCAAGAGG
CCTGTGCAAATAGCAGCGGTGGTTACCACCGCATCAACGTTTATGAGATTCTCAAGGCAGAA
GGATCCCCTGGCAGTGATCCGATCACGCGATTGCTGGACACAAAACTGGTGCACCACAACGT
GAGCAAGTGGGAGAGCTTTGACGTCAGTCCTTCTGTCATGAGATGGACCGTACAGGGGCAGC
CCAACCATGGGTTTATGGTGGAAGTCATTCACTTGGAGCAGGAGTGCAGACACTCAAAGCGA
CATGTCAGGATCAGTCGGTCCTTGCACCAAGATGAAGAAAGCTGGCCTCAGATGAGGCCTTT
GTTGGTAACGTTTAGCCACAATGGCAAAGGACATACTCTTGAGAAAAGAGTGAGGCGTCAGG
CCAAACACAAGCAGAAGAAAAGGCACAGGTCGAGCTGCAAGCGGCATCCTTTATACGTGGAT
TTCAGTGACGTGGGGTGGAATGACTGGATAGTGGCACCTCCAGGATATCACGCCTTTTACTG
CCAAGGGGAGTGTCCCTTTCCATTGGCAGATCACTTGAACTCAACGAACCATGCCATTGTGC
AGACATTGGTAAACTCTGTCAATGCAAACATTCCCAGGGCATGCTGCGTCCCAACGGACCTC
AGTCCCATCTCAATGCTTTATCTTGATGAATACGACAAAGTTGTATTAAAGAACTACCAAGA
TATGGTTGTGGAAGGTTGTGGATGTCGT

>*Scyliorhinus torazame Bmp4*
ATGATTCCTGGTAACCGAATGCTGATGGTAATTTTATTATGCCAAGTCTTACTGGGAGGTAA
TGCTAGTCTGATACCAGAGGAAGGGAGGAAAAAGTTCGCTGAGCAAAACCAGGCGGGTGGAC
GTCGCTCTCCGCAAAACCATGAACTGCTACGCGAATTCGAGGCCACTTTACTCAACATGTTC
GGGCTGCAGAGGCGCCCGCAGCCCAGCAAGTCTGCGGTCGTGCCCCAGTACATGCTGGATCT
CTACAGGCTGCAGTCCGGGGACGAGATCACCCACGACATTAGCTTCGAGTATCCCGAGAGAT
CCACGAGCCGGGCCAACACCGTGAGGAGCTTTCACCACGAAGAACACTTGGAGCTGATGCCA
GGGCAGAGGGAAGACACCCAGCTTCGCTTTGTCTTTAATATCAGCGTGGTGCCCGAGAACGA
GGTGCTCTCTTCTGCGGAGCTGCGGCTGTACCGGGAGCAGATCGACAGCGTTTGGAGCAGCG

AGGAGGATGGGTTCCATCGCATCAACGTTTACGAGATCATGAGAGCCCCCAGCGAGAAGGGA
GGGCTGATCAGCAGGCTCCTCGACACCAGGCTGGTGCACCAGAACAGGACGCGCTGGGAGAG
CTTTGACGTGAGCCCGGCGGTGCTGAGGTGGACCATGCAGAGAGAGCCCAACCACGGCCTGG
CCGTCGAGGTGATCCACCTGAACGAGACTACCAAGAGGCGGCACGTCCGGATTAGCAGGTCG
CTGCACCAAAGGCACGCCGGCGACTCGTCCCAGCTCAGGCCTTTACTTGTAACTTTCAGCCA
CGACGGCAAGGGACACGCTCTCACGCGGAGGGTGAAACGGAGCAGCAAGGGGCAGAGGCGCA
AGAAGAACAAATCTCACTGCAGGAGGCACTCCCTCTACGTGGATTTCAGCGACGTGGGCTGG
AACGACTGGATCGTCGCGCCGCCCGGTTACCAAGCTTTTTACTGCCACGGGGACTGCCCCTT
CCCACTGGCGGACCACCTGAACTCGACGAACCACGCCATCGTGCAGACACTCGTCAATTCGG
TGAACTCCAACATCCCCAAGGCTTGCTGCGTCCCCACAGAACTCAGCCCCATCTCAATGCTT
TACTTGGACGAGTATGACAAAGTTGTGCTGAAAAACTATCAGGAGATGGTCGTGGAGGGATG
TGGCTGCCGC

>*Scyliorhinus torazame Bmp16*
ATGCTCCCTGCTAGCTTTCTACAGATGGTCGTGCTGTCTGCCCGGCTCCTGGTCGCTGGAGA
GATCAACTCGTCCTCTGAGGGAGGGAGGAGGGCATCTTCGAGTGCCAGCCGGGAGGAAGGTG
CCTCCCGCTCCTCGCCCGATGCGGAGCTGGTCCACACCCTCCGCCGGACGCTGCTGGGCAGG
TTTGGCCTGAGCCGCCTCCCCAAGCCAGGCCTGGCCGCCGTCGTCCCTCAGTACATGCGTGA
CCTGTACCACCTCCACACCGGCGATCACGACAACATCCAGGGCTCCAGCTTCGCCTTCCTGG
AGCGGCACGCGGGCGACGCCAACACAGTCCGGAGCTTCCATCATCTGGAACACGTCCAGGGG
GCGCCCCATTCAGAGGGAGGAAATTTCCACCAGATTGTCTTCAACCTGACCAACATTCCCAA
CCACGAGCGAGTCACTTCGGCCGAGCTGCGTTTATTCAGGGAAGATAATGGTGGAGCACGTC
CTGGGCTGGAAGCGGTCGCCGTCTACGAACTGCGGGGGCCCCCAGGCGGCCAGGTGGGGCGC
CGGCTGCTGGAGAGGAGGCTACTGCCCGCCAACCGCACCCAATGGGAAAGCTTCAACGTGGG
CCCGACTGTGGCCGGCTCCAGGAAGGGGCGCGGGGGCCTCGTCACCTTCCTGGTCGAGTGGC
CGCCGGGGCGGCGGAGGGTGGGCAGGTCGCTGTGGGGGCGGCCGGCCGGCCAGCCGGGTCGC
AGGCCGCTCCTGGTCACCTACGGGCAGGACGGCAGCGGGAGGGCCCTGGGCCCCAGGGGCAA
GCGGCCCGGCGGGCGGCGGAGGACGCGGAAGAGGAGCCGGGGCCACAGCCGCTGCCGGAGGC
GGCCGCTCTACGTGGACTTTGAGCGGGTGGGCTGGACGGAGTGGATCATTGCCCCCCGTGGC
TACAACGCCTTCTACTGCCAGGGCGAGTGCCGCTTCCCCTTGGCCGACCACATGAACTCCTC
CAGCCATGCCATCGTGCAGACGCTGGTGAACTCTGTCAACACCAACATTCCCAGGGCCTGCT
GCGTGCCAACCGAGCTGAGCCCAGTGGCCATGCTCTACTTGGACGACGATGACAGGGTCGTC
CTGAAGAATTACCAGGAGATGGTTGTCGAAGGGTGTGGATGCCGC
